# Long-term effects of empagliflozin on excitation-contraction-coupling in human induced pluripotent stem cell cardiomyocytes

**DOI:** 10.1007/s00109-020-01989-6

**Published:** 2020-10-09

**Authors:** Steffen Pabel, Florian Reetz, Nataliya Dybkova, Orr Shomroni, Gabriela Salinas, Julian Mustroph, Karin P. Hammer, Gerd Hasenfuss, Nazha Hamdani, Lars S. Maier, Katrin Streckfuss-Bömeke, Samuel Sossalla

**Affiliations:** 1 Department of Internal Medicine II, University Medical Center Regensburg, Franz-Josef-Strauß-Allee 11, 93053 Regensburg, Germany; 2 Clinic for Cardiology & Pneumology, Georg-August University Goettingen, and DZHK (German Center for Cardiovascular Research), Robert-Koch-Str. 40, 37075 Goettingen, Germany; 3 NGS-Integrative Genomics (NIG) Institute Human Genetics (O.S., G.S.), University Medical Center Goettingen, Georg-August University, Goettingen, Germany; 4 Institute of Physiology, Ruhr University Bochum, Bochum, Germany

**Keywords:** Empagliflozin, EC-coupling, Electrophysiology, iPSC-CM

## Abstract

**Abstract:**

The SGLT2 inhibitor empagliflozin improved cardiovascular outcomes in patients with diabetes. As the cardiac mechanisms remain elusive, we investigated the long-term effects (up to 2 months) of empagliflozin on excitation-contraction (EC)-coupling in human cardiomyocytes derived from induced pluripotent stem cells (iPSC-CM) in a blinded manner. IPSC from 3 donors, differentiated into pure iPSC-CM (4 differentiations), were treated with a clinically relevant concentration of empagliflozin (0.5 μmol/l) or vehicle control. Treatment, data acquisition, and analysis were conducted externally blinded. Epifluorescence microscopy measurements in iPSC-CM showed that empagliflozin has neutral effects on Ca^2+^ transient amplitude, diastolic Ca^2+^ levels, Ca^2+^ transient kinetics, or sarcoplasmic Ca^2+^ load after 2 weeks or 8 weeks of treatment. Confocal microscopy determining possible effects on proarrhythmogenic diastolic Ca^2+^ release events showed that in iPSC-CM, Ca^2+^ spark frequency and leak was not altered after chronic treatment with empagliflozin. Finally, in patch-clamp experiments, empagliflozin did not change action potential duration, amplitude, or resting membrane potential compared with vehicle control after long-term treatment. Next-generation RNA sequencing (NGS) and mapped transcriptome profiles of iPSC-CMs untreated and treated with empagliflozin for 8 weeks showed no differentially expressed EC-coupling genes. In line with NGS data, Western blots indicate that empagliflozin has negligible effects on key EC-coupling proteins. In this blinded study, direct treatment of iPSC-CM with empagliflozin for a clinically relevant duration of 2 months did not influence cardiomyocyte EC-coupling and electrophysiology. Therefore, it is likely that other mechanisms independent of cardiomyocyte EC-coupling are responsible for the beneficial treatment effect of empagliflozin.

**Key messages:**

- This blinded study investigated the clinically relevant long-term effects (up to 2 months) of empagliflozin on cardiomyocyte excitation-contraction (EC)-coupling.
- Human cardiomyocytes derived from induced pluripotent stem cells (iPSC-CM) were used to study a human model including a high repetition number of experiments.
- Empagliflozin has neutral effects on cardiomyocyte Ca^2+^ transients, sarcoplasmic Ca^2+^ load, and diastolic sarcoplasmic Ca^2+^ leak.
- In patch-clamp experiments, empagliflozin did not change the action potential.
- Next-generation RNA sequencing, mapped transcriptome profiles, and Western blots of iPSC-CM untreated and treated with empagliflozin showed no differentially expressed EC-coupling candidates.

## Introduction

The antidiabetic drug empagliflozin, which inhibits the sodium-dependent glucose cotransporter 2 (SGLT2) in the kidney, has been shown to reduce cardiovascular mortality, all-cause mortality, and hospitalization rates for heart failure (HF) in patients at high cardiovascular risk and type 2 diabetes [1]. These beneficial effects have been also confirmed for other SGLT2 inhibitors [2, 3] and, importantly, also in a diabetes-independent manner in patients with HF [4]. Moreover, recent clinical data indicate that this improved cardiovascular outcome might not be dependent on modulation of cardiovascular risk factors as the effects occur already after a few months [5]. Therefore, the question arises whether empagliflozin has direct cardiac effects. Despite a growing number of studies investigating the cardiovascular effects of empagliflozin, the underlying mechanisms are still under debate and remain controversial. Importantly, direct myocardial effects of empagliflozin on cardiac metabolism [6], contractility [7, 8], and cardiomyocyte Ca^2+^ and Na^+^ homeostasis [9, 10] have been proposed. One potential mechanisms might be an inhibitory effect of empagliflozin on the Na^+^/H^+^ exchanger (NHE) causing a reduction in myocardial cytoplasmic Na^+^ and Ca^2+^ and increased mitochondrial Ca^2+^ [10, 11]. As Ca^2+^ cycling fundamentally determines cardiac excitation-contraction (EC)-coupling and thereby cardiac function, changes in Ca^2+^ homeostasis are associated with pathological cardiac remodeling and HF [12]. Therefore, the evaluation whether empagliflozin may affect cardiomyocyte EC-coupling is of importance to understand its potential cardiac effects and for further translational investigation. However, the role of empagliflozin for cardiomyocyte EC-coupling is not fully elucidated, and previous reports are limited by small n-numbers. As the systemic effects of empagliflozin may indirectly affect the myocardium, the investigation of the direct cardiac effects of empagliflozin is thus only possible in in vitro models. However, isolated adult cardiomyocytes (CM), as investigated previously [7, 9, 10], are not suitable for chronic culture and treatment protocols according to the clinical setting, where the beneficial effects of empagliflozin became apparent after ~ 2 months of treatment [1].

The aim of this study was to fundamentally investigate the clinically relevant direct long-term effects of empagliflozin on human cardiomyocyte EC-coupling and electrophysiology in a standardized and blinded manner by using human-induced pluripotent stem cell cardiomyocytes (iPSC-CM). Based on the clinical data, iPSC-CM were chronically treated for 2 and 8 weeks with empagliflozin (0.5 μmol/l), and EC-coupling and electrophysiology were studied by blinded investigators.

## Methods

### IPSC-CM and treatment

All procedures were performed according to the Declaration of Helsinki and were approved by the local ethics committee. Informed consent was obtained from all tissue donors. IPSCs were cultured feeder-free and adherent by cultivating on Geltrex-coated cell culture dishes in the presence of chemically defined medium E8 (Life Technologies). Cardiac differentiation of iPSCs was performed by sequential targeting of the WNT pathway as described previously [13]. Briefly, undifferentiated iPSCs were cultured as a monolayer on Geltrex-coated 12-well dishes to a confluence of 85–95%. Medium was changed to cardio differentiation medium composed of RPMI 1640 medium (Thermo Fisher Scientific) supplemented with 0.02% L-ascorbic acid 2-phosphate (Sigma Aldrich) and 0.05% albumin (Sigma Aldrich) including the GSK3 inhibitor CHIR99021 (4 μmol/L, Millipore) (d0). After 48 h, medium was changed to fresh media supplemented with 5 μmol/L of the inhibitor of Wnt production-2 (IWP2, Millipore) for 2 days. From day 10 on, the cells were cultured in cardio culture medium (RPMI 1640 medium supplemented with 2 mmol/L l-glutamine and 2% B27 with insulin (Life Technologies)), with a medium change every 2–3 days. CM were purified using metabolic selection for 2–4 days by using 4 mmol/L lactate as carbon source after 20–40 days of differentiation [14] and studied on day 90 after initiation of differentiation except when mentioned otherwise. Following differentiation, purity of iPSC-CM was determined by flow analysis (> 90% cardiac TNT^+^) or by morphology. Four- to 5-week-aged iPSC-CM were treated with 0.5 μmol/l empagliflozin for 2 or 8 consecutive weeks, which corresponds to the clinical relevant plasma concentration [15, 16]. All investigators were blinded by an external independent instance during treatment, data acquisition, data analysis, and statistical testing. All analyses were performed in iPSC-CM with or without empagliflozin treatment. Four differentiation experiments of different iPSC lines from 3 healthy probands (female) were used.

### Epifluorescence microscopy (Ca^2+^ transients and sarcoplasmic reticulum Ca^2+^ content)

IPSC-CMs were loaded for 15 min with the ratiometric Ca^2+^ dye Fura-2 AM (5 μmol/l). Solution was replaced by Tyrode’s solution (in mmol/l: KCl 4, NaCl 140, MgCl_2_ 1, HEPES 10, glucose 10, CaCl_2_ 1.25, pH 7.4, NaOH), and the respective agent (empagliflozin/vehicle control) and cells were left to incubate for another 15 min to ensure complete deesterification of intracellular Fura-2 and allow cellular rebalance of Ca^2+^ cycling properties. The Tyrode’s solution was again replaced before measurements were started. Fura-2 fluorescence ratio was calculated using alternating excitation at 340 nm and 380 nm. The emitted fluorescence was collected at 510 nm. Measurements were performed with a fluorescence detection system (ION OPTIX Corp.). Ca^2+^ transients were recorded at steady-state conditions under constant field stimulation (30 V, 10 ms) during increasing frequencies (0.25, 0.5, 1 Hz) at room temperature. Sarcoplasmic Ca^2+^ content was assessed by caffeine application (10 mmol/L) after stopping field stimulation (0.25 Hz) to induce a complete sarcoplasmic reticulum Ca^2+^ release. The recorded Ca^2+^ transients were analyzed with the software IONWizard (ION OPTIX Corp.).

### Confocal microscopy (diastolic Ca^2+^ sparks)

IPSC-CM were incubated with Fluo-4 AM (10 μmol/l) for 15 min before cells were incubated with Tyrode’s solution for another 15 min as described above. Ca^2+^ spark measurements were performed with a laser scanning confocal microscope (LSM 7 Pascal, Zeiss) at room temperature. Fluo-4 was excited by an argon ion laser (488 nm), and emitted fluorescence was collected through a 505-nm long-pass emission filter. Fluorescence images were recorded in the line scan mode with 512 pixels per line (width of each scan line: 35.4 μm) and a pixel time of 0.64 μs. One image consists of 10,000 unidirectional line scans, equating to a measurement period of ~ 6.9 s. Experiments were conducted at resting conditions after loading the SR with Ca^2+^ by repetitive field stimulation for 10 s (at 0.5 Hz, 30 V, 5 ms). Diastolic Ca^2+^ sparks were analyzed with the program SparkMaster for ImageJ. The Ca^2+^ spark frequency of each cell resulted from the number of sparks normalized to cell width and scan rate (100 μm^−1^*s^−1^). The Ca^2+^ spark size was calculated as the product of spark amplitude, duration, and width. To compute the full Ca^2+^ leak per cell, Ca^2+^ spark size was multiplied with Ca^2+^ spark frequency [17].

### Action potential measurements

IPSC-CM were incubated with Tyrode’s solution and the respective agent (empagliflozin/vehicle control) for 15 min before measurements were started. For action potential recordings, whole-cell patch-clamp technique was used (current clamp configuration, HEKA electronics) [17, 18]. Microelectrodes (2–3 MΩ) were filled with (in mmol/L) 122 K-aspartate, 8 KCl, 10 NaCl, 1 MgCl2, 10 HEPES, and 5 Mg-ATP (pH 7.2, KOH). Action potentials were continuously elicited by square current pulses of 0.5–1 nA amplitude and 1–5 ms duration at 0.25, 0.5, 1, and 2 Hz. Access resistance was < 15 MΩ after rupture. Fast capacitance was compensated in cell-attached configuration. Membrane capacitance and series resistance were compensated after patch rupture. Signals were filtered with 2.9 and 10 kHz Bessel filters and recorded with an EPC10 amplifier (HEKA Elektronik) [19]. All experiments were conducted at room temperature.

### RNA sequencing (RNA-seq) and bioinformatics

Differential gene expression was obtained by use of RNA sequencing performed on an Illumina HighSeq-4000 platform and bioinformatics. Total RNA was isolated from 3-month-old iPSC-CM from 2 controls with 2 differentiation each (*n* = 4 samples) treated and non-treated with empagliflozin using standard protocols (Promega).

Quality and integrity of RNA were assessed with the Fragment Analyzer from Advanced Analytical by using the standard sensitivity RNA Analysis Kit (DNF-471). All samples selected for sequencing exhibited an RNA integrity number over 8. RNA-seq libraries were performed using 150 ng total RNA of a nonstranded RNA-seq, massively parallel mRNA sequencing approach from Illumina (TruSeq stranded total RNA Library Preparation, Illumina). Libraries were prepared on the automation (Beckman Coulter’s Biomek FXP workstation). For accurate quantitation of cDNA libraries, a fluorometric based system, the QuantiFluor™dsDNA System from Promega, was used. The size of final cDNA libraries was determined by using the dsDNA 905 Reagent Kit (Fragment Analyzer from Advanced Bioanalytical) exhibiting a sizing of 300 bp in average. Libraries were pooled and sequenced on the Illumina HiSeq 4000 (SE; 1 × 50 bp; 30–35 Mio reads/sample).

Sequence images were transformed with Illumina software BaseCaller to BCL files, which was demultiplexed to fastq files with bcl2fastq v2.17.1.14.

#### Raw read and quality check

Sequence images were transformed with Illumina software BaseCaller to BCL files, which was demultiplexed to fastq files with bcl2fastq v2.20.0.422. The sequencing quality was asserted using FastQC (Andrews, S. (2014). FastQC A Quality Control tool for High Throughput Sequence Data. http://www.bioinformatics.babraham.ac.uk/projects/fastqc/)(version 0.11.5).

#### Mapping and normalization

Sequences were aligned to the reference genome *Homo sapiens* (hg38 version 96, https://www.ensembl.org/Homo_sapiens/Info/Index) using the STAR aligner (Dobin, Alexander, et al. “STAR: ultrafast universal RNA- seq aligner.” Bioinformatics 29.1 (2013): 15–21.) (version 2.5.2a) allowing for 2 mismatches within 50 bases. Subsequently, read counting was performed using featureCounts (Liao, Yang, Gordon K. Smyth, and Wei Shi. “featureCounts: an efficient general purpose program for assigning sequence reads to genomic features.” Bioinformatics 30.7 (2013): 923–930.) (version 1.5.0-p1). Read counts were normalized in the R/Bioconductor environment (version 3.6.1, www.bioconductor.org) using the DESeq2 R package (Love, Michael I., Simon Anders, and Wolfgang Huber. “Moderated estimation of fold change and dispersion for RNA-seq data with DESeq2.” Genome biology 15.12 (2014): 550.) version 1.24.0, where normalization was done by calculating the size factors for each sample and dividing by them.

### Western blots

Pellets of iPSC-CMs were snap-frozen in liquid nitrogen and stored at − 80 °C. Protein lysates were prepared in cell lysis buffer containing (mmol/L) 20 Tris-HCl, 200 NaCl, 20 NaF, 1 Na_3_VO_4_, 1 DTT, 1% Triton X-100 (pH 7.4), and complete protease and phosphatase inhibitor cocktails (Roche Diagnostics, Germany). About 20–25 μg of protein was loaded with SDS sample buffer (30 min, 37 °C in 2% beta-mercaptoethanol). The protein samples were separated on 5/10/15% SDS-PAGE, transferred, and probed with the following primary antibodies: mouse monoclonal anti-GAPDH (1:30000, BTMC-A473-9, BIOTREND), rabbit polyclonal anti-RyR2 (1:15000, HPA020028, Sigma), mouse monoclonal anti-PLB (1:5000, MA3-922, Thermo Scientific), rabbit polyclonal anti-pCaMKII (1:1000, PA5–36838, Thermo Scientific), rabbit polyclonal anti-CaMKII (1:5000, PA5-22168, Thermo Scientific), mouse monoclonal anti-NCX (1:1000, R3F1, Swant), and mouse monoclonal anti-SERCA (1:20000, MA3–919, Thermo Scientific). Secondary antibodies included HRP-conjugated goat anti-rabbit and goat anti-mouse (1:30000, 111-035-144 and 115-035-062, respectively, Jackson Immunoresearch). ImmobilonTM Western Chemiluminescent HRP Substrate (Millipore) was used for the chemiluminescent detection. The intensity of individual bands from Western blots was quantified using Image Studio Lite software, normalized to GAPDH, and then shown relative to the control group value.

### Statistics

All data are presented as mean values ± SEM. For statistical testing of longitudinal data including more than two groups, 2-way ANOVA corrected for multiple comparisons by the Sidak method was used. For comparison of two groups, Student’s *t* test was applied. Analysis was performed using GraphPad Prism 8. *P* values were two-sided and considered statistically significant if *P* < 0.05.

## Results

### Effects of empagliflozin on systolic Ca^2+^ transients and SR Ca^2+^ load

**Fig. 1:**
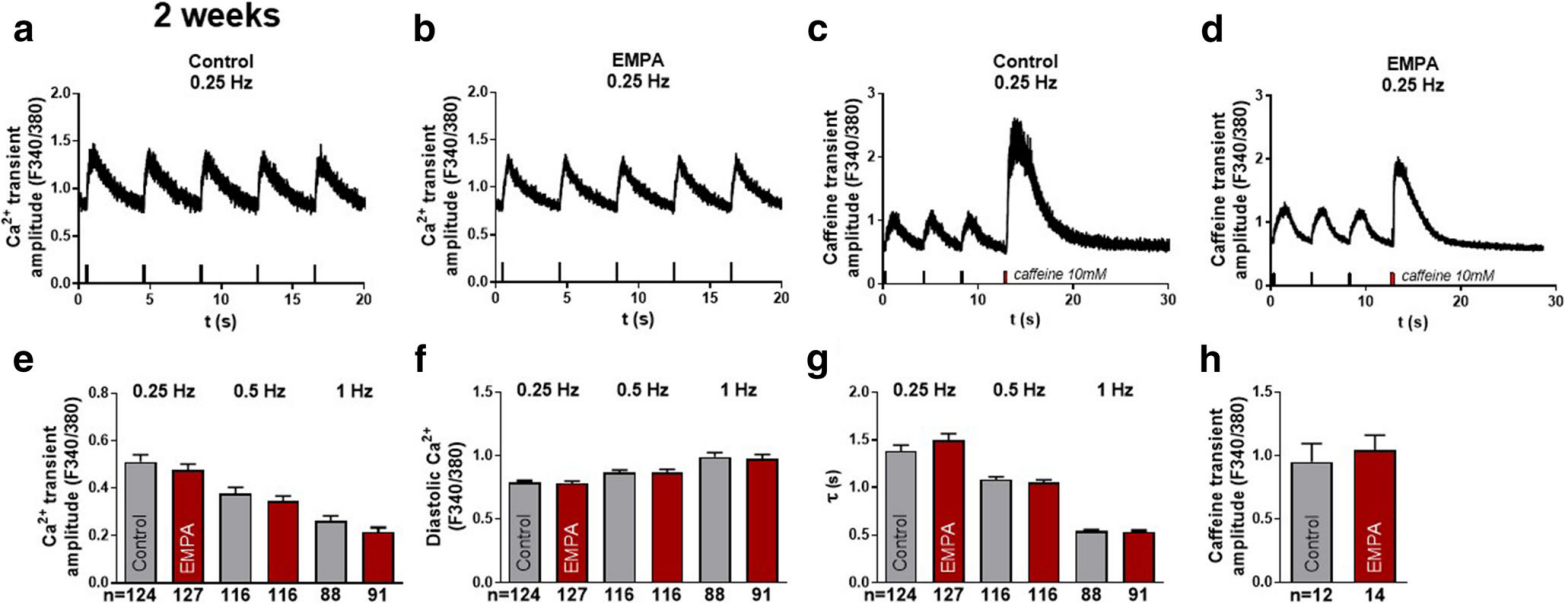
Systolic Ca^2+^ transients and SR Ca^2+^ load (epifluorescence microscopy, Fura-2 AM 5 μM) of human-induced pluripotent stem cell cardiomyocytes (iPSC-CM) after 2 weeks of treatment with either vehicle control (control) or 0.5 μmol/l empagliflozin (EMPA). (**a**–**b**) Original representative stimulated Ca^2+^-transient recordings at 0.25 Hz after 2 weeks of treatment and (**c**–**d**) Ca^2+^ transients recorded during application of 10 mM caffeine indicating SR Ca^2+^ load. (**e**) Mean data during increasing stimulation frequencies (0.25, 0.5, and 1 Hz) for systolic Ca^2+^-transient amplitude (**f**), diastolic Ca^2+^ levels, and (**g**) exponential decay time of Ca^2+^ transients (τ). (**h**) Mean data for caffeine-transient amplitude. The sample sizes of iPSC-CM are depicted below each column. Groups were statistically compared using two-way ANOVA with Sidak’s test for multiple comparisons or Student’s *t* test (**h**)

**Fig. 2:**
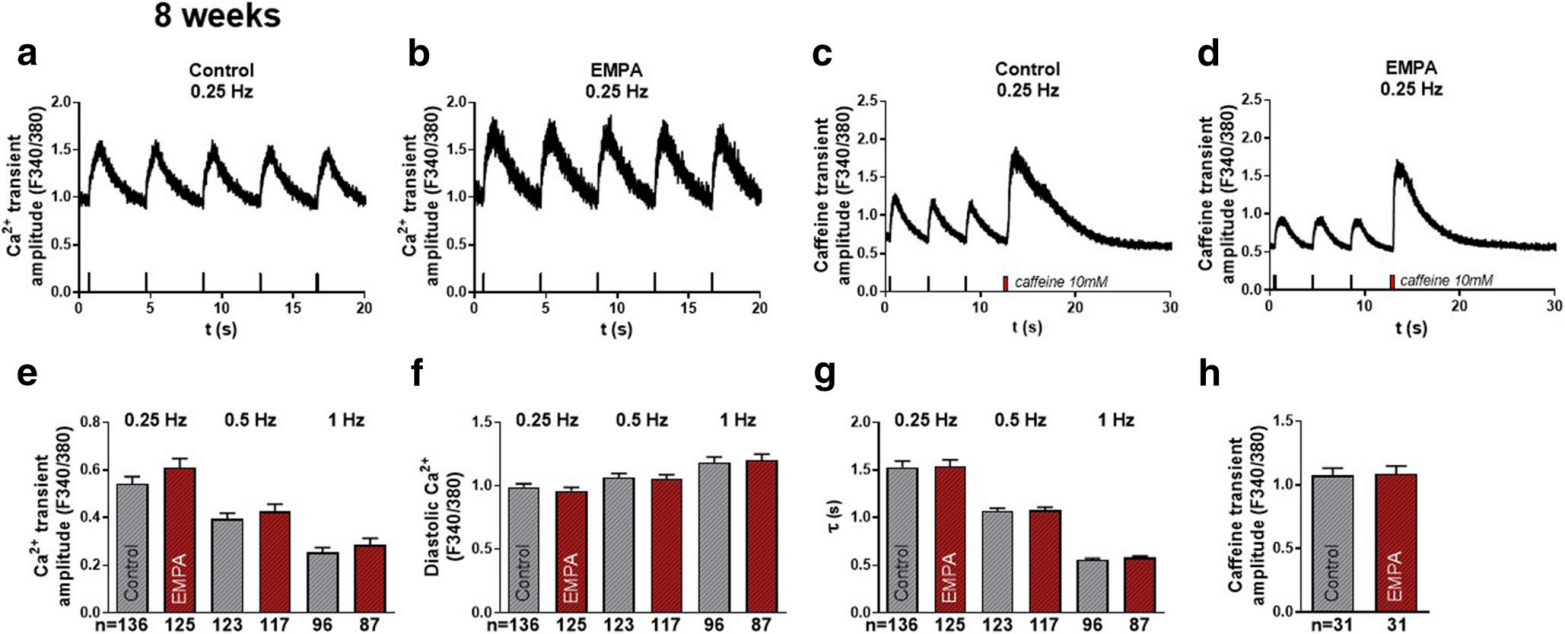
Systolic Ca^2+^ transients and SR Ca^2+^ load (epifluorescence microscopy, Fura-2 AM 5 μM) of human-induced pluripotent stem cell cardiomyocytes (iPSC-CM) after 8 weeks of treatment with either vehicle control (control) or 0.5 μmol/l empagliflozin (EMPA). (**a**–**b**) Original representative stimulated Ca^2+^-transient recordings at 0.25 Hz of 8 weeks treated iPSC-CMs and (**c**–**d**) caffeine-induced transients indicating SR Ca^2+^ content. (**e**) Mean data during increasing stimulation frequencies (0.25, 0.5, and 1 Hz) for systolic Ca^2+^-transient amplitude (**f**), diastolic Ca^2+^ levels, and (**g**) exponential decay time of Ca^2+^ transients (τ). (**h**) Mean data for caffeine-transient amplitude. The sample sizes of iPSC-CM are provided below the respective column. For statistical analysis, two-way ANOVA with Sidak’s test for multiple comparisons or Student’s *t* test (**h**) was used

As empagliflozin was shown to (acutely) influence Ca^2+^ cycling [9, 10], we investigated the chronic effects (2 and 8 weeks) of empagliflozin on stimulated Ca^2+^ transients using epifluorescence microscopy (Fura-2). Caffeine application was used to assess the SR Ca^2+^ load. After 2 weeks of treatment, empagliflozin did not change systolic Ca^2+^ transient amplitude, diastolic Ca^2+^ levels, or the Ca^2+^ elimination kinetics during increasing stimulation frequencies (0.25, 0.5, 1 Hz) in iPSC-CM (*n* = 127, 116, 91 cells) compared with control (*n* = 124, 116, 88 cells, Fig. 1 a–b and e–g). Moreover, SR Ca^2+^ load (caffeine-transient amplitude) was not altered in the empagliflozin group (*n* = 14 cells) compared with the control group (*n* = 12 cells, Fig. 1c–d and h). After 8 weeks of treatment, which corresponds to the time of onset of the clinical effects of empagliflozin, Ca^2+^ transient amplitude in control iPSC-CM (F340/380: 0.54 ± 0.03, *n* = 136 cells) was not significantly different compared with the empagliflozin-treated group (0.61 ± 0.04, *n* = 125 cells) at 0.25 Hz and during increasing frequencies up to 1 Hz (Fig. 2a–b and e). Also, diastolic Ca^2+^ levels (F340/380 at 0.25 Hz: control: 0.99 ± 0.02, empagliflozin: 0.96 ± 0.03) and Ca^2+^ elimination (tau (s), 0.25 Hz; control: 1.53 ± 0.07 s; empagliflozin: 1.54 ± 0.07 s) were not changed (Fig. 2f–g). In line with that, SR Ca^2+^ load did not differ between control (*n* = 31 cells) and empagliflozin (*n* = 31 cells) after 8 weeks of treatment (Fig. 2c–d and h). Thus, chronic treatment with empagliflozin has neutral effects on cardiomyocyte systolic and diastolic Ca^2+^ cycling in iPSC-CM.

### Influence of chronic empagliflozin treatment on diastolic Ca^2+^ release in iPSC-CM

**Fig. 3:**
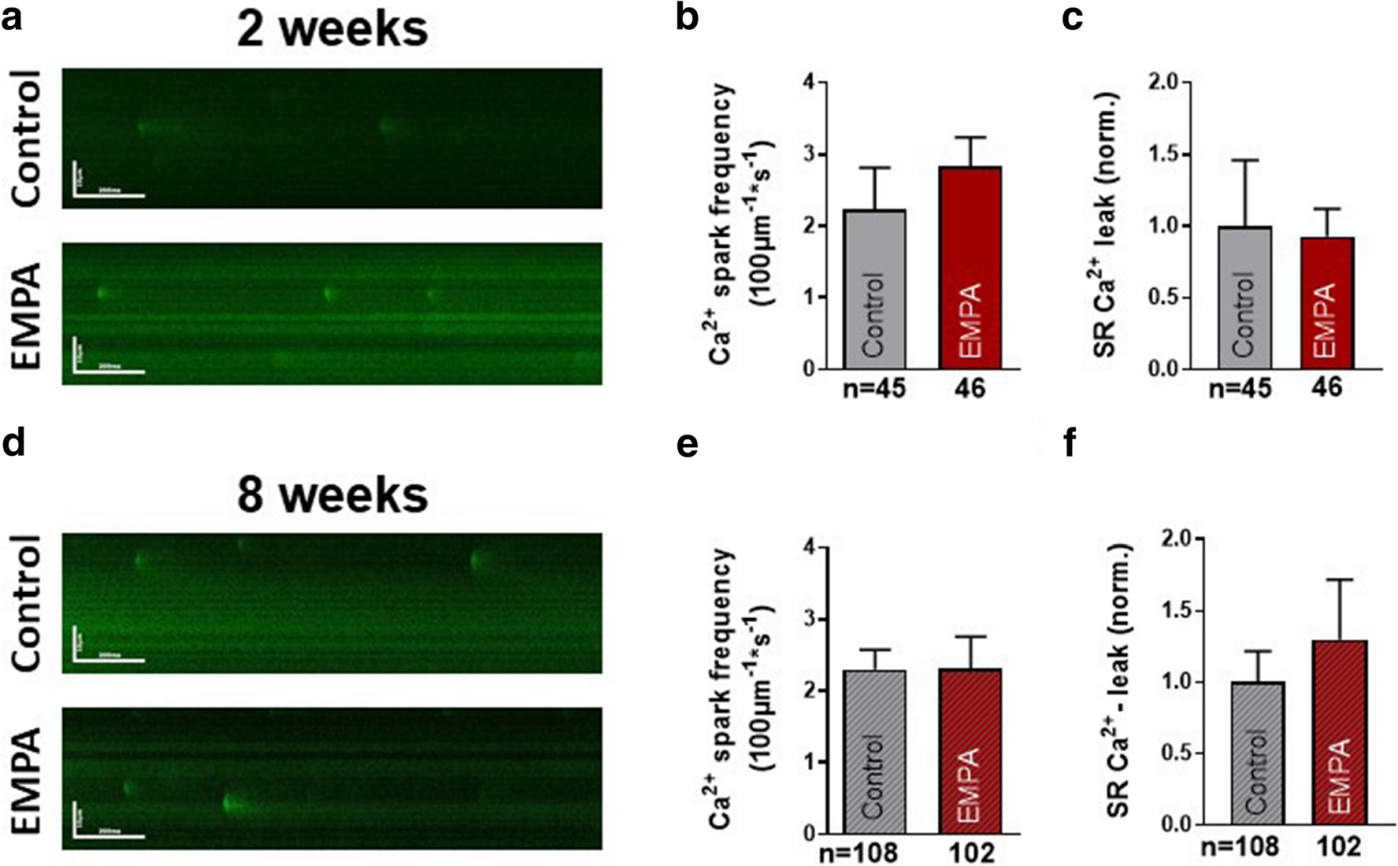
Diastolic sarcoplasmic reticulum Ca^2+^ release (confocal microscopy, Fluo-4 AM 10 μM) of human-induced pluripotent stem cell cardiomyocytes (iPSC-CM) after 2 and 8 weeks of treatment with either vehicle control (control) or 0.5 μmol/l empagliflozin (EMPA). (**a**) Representative original confocal line scans showing diastolic Ca^2+^ sparks in iPSC-CM after 2 and (**d**) after 8 weeks treatment with empagliflozin compared with vehicle control. (**b**) Mean data of diastolic Ca^2+^ spark frequency and (**c**) the total calculated diastolic Ca^2+^ leak (normalized to vehicle control) after 2 weeks and (**e**–**f**) 8 weeks of empagliflozin treatment. The sample sizes of iPSC-CM are presented below the respective column. Groups were statistically tested using Student’s *t* test

Diastolic Ca^2+^ release via leaky RyR2 is a key mechanism for contractile dysfunction and arrhythmias in HF and other cardiac diseases [12]. Previous investigation suggested that empagliflozin reduces diastolic Ca^2+^ release in HF CM [9]. We therefore investigated the direct long-term effects of empagliflozin on diastolic SR Ca^2+^ release using confocal microscopy (Fluo-4). After 2 weeks of treatment with either empagliflozin (*n* = 46 cells) or vehicle control (*n* = 45), empagliflozin showed no effects on diastolic Ca^2+^ spark frequency (Fig. 3a–b) and the total calculated diastolic Ca^2+^ leak (Fig. 3c) in iPSC-CM. After 8 weeks of treatment, which corresponds to the time where the clinical effects of empagliflozin became apparent, diastolic Ca^2+^ spark frequency in empagliflozin-treated iPSC-CM (*n* = 102 cells) was determined at 2.3 ± 0.4/100 μm/s, but was not changed compared with iPSC-CM treated with vehicle control (2.3 ± 0.3/100 μm/s, *n* = 108, Fig. 3d–e). Accordingly, the total calculated diastolic Ca^2+^ leak did not differ in iPSC-CM treated with either empagliflozin or vehicle control (Fig. 3f).

### Effects of empagliflozin on cardiac action potentials

**Fig. 4:**
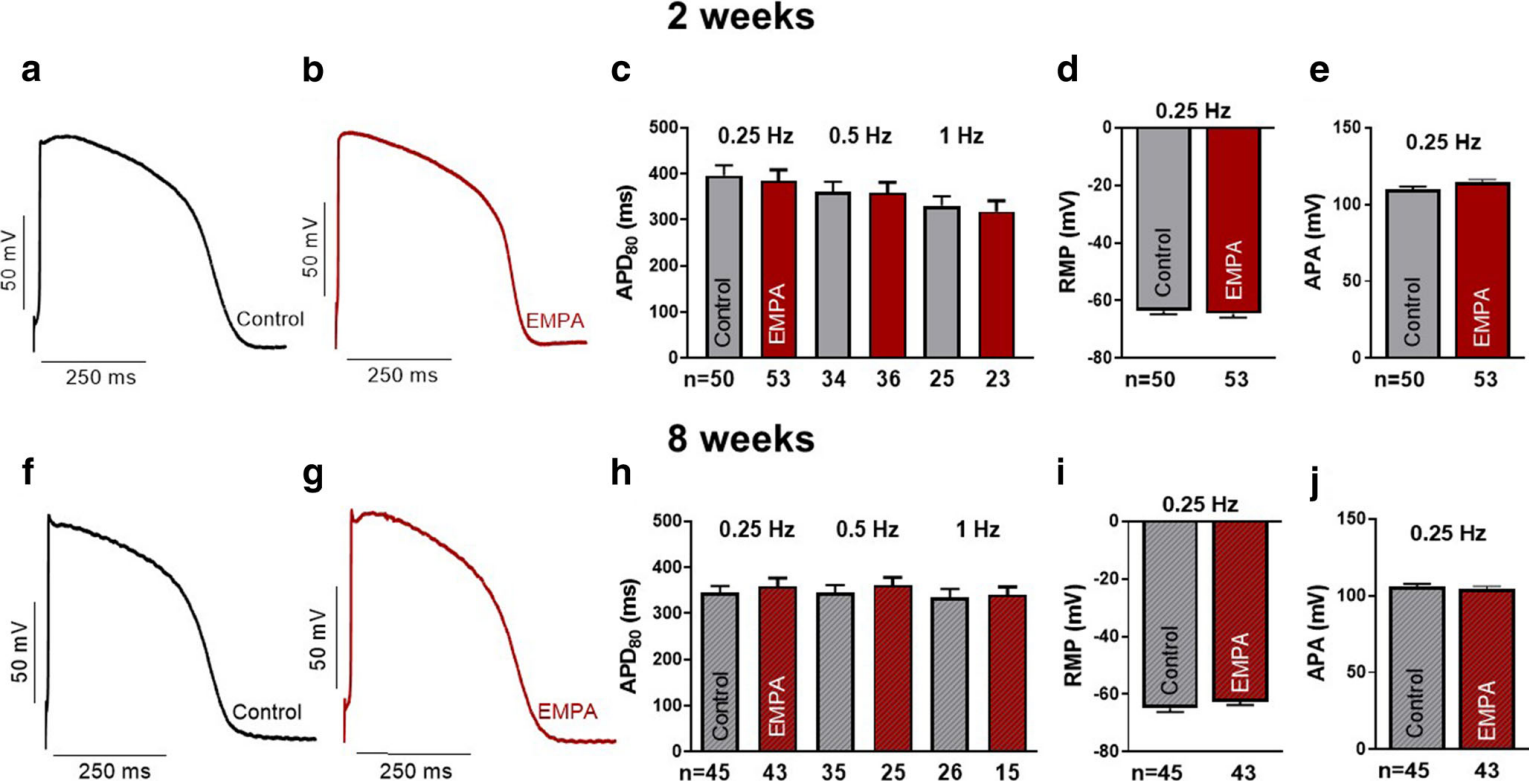
Action potentials (patch-clamp technique) of human-induced pluripotent stem cell cardiomyocytes (iPSC-CM) after treatment with either vehicle control (Control) or 0.5 μmol/l empagliflozin (EMPA). (**a**) Representative action potential recordings (0.25 Hz) of human iPSC-CM treated for 2 weeks with vehicle control or (**b**) empagliflozin. (**c**) Effects of 2 weeks treatment with control or empagliflozin on action potential duration at 80% (APD_80_), (**d**) resting membrane potential (RMP) and (**e**) action potential amplitude (APA). (**f**) Original action potential recordings (0.25 Hz) of 8 weeks treated human iPSC-CM with either control or (**g**) empagliflozin. (**h**) Mean data for APD_80_, (**i**) RMP, and (**j**) APA of iPSC-CM after 8 weeks of treatment with control or empagliflozin. The sample sizes of iPSC-CM are provided below the respective column. For statistical testing, two-way ANOVA with Sidak’s test for multiple comparisons (**c** and **h**) or Student’s *t* test was used

The action potential is a critical component of cardiomyocyte electrophysiology and adverse electric remodeling in HF. To study potential direct effects of chronic empagliflozin treatment on the action potential, we investigated stimulated action potentials in iPSC-CM using the patch-clamp technique. In iPSC-CM treated for 2 weeks with empagliflozin (*n* = 53 at 0.25 Hz), we observed no differences in action potential duration at 80% repolarization (APD80), resting membrane potential, or action potential amplitude compared with control iPSC-CM (*n* = 50, Fig. 4a–e). After 8 weeks of treatment, APD80 at 0.25 Hz was 344.4 ± 15.4 ms in control-treated cells (*n* = 45). APD80 (358.5 ± 18.5 ms) in empagliflozin-treated iPSC-CM (*n* = 43) was not significantly different compared to control (Fig. 4f–h). Also, resting membrane potential (control: − 62.4 ± 1.0 mV; empagliflozin: − 64.3 ± 1.1 mV) and action potential amplitude (control: 103.4 ± 1.7 mV; empagliflozin: 104.4 ± 1.8 mV) were not changed by empagliflozin after 8 weeks of treatment. In conclusion, empagliflozin showed neutral effects on cellular action potentials.

### Effects of chronic empagliflozin treatment on gene expression profiles of human iPSC-CM

**Fig. 5:**
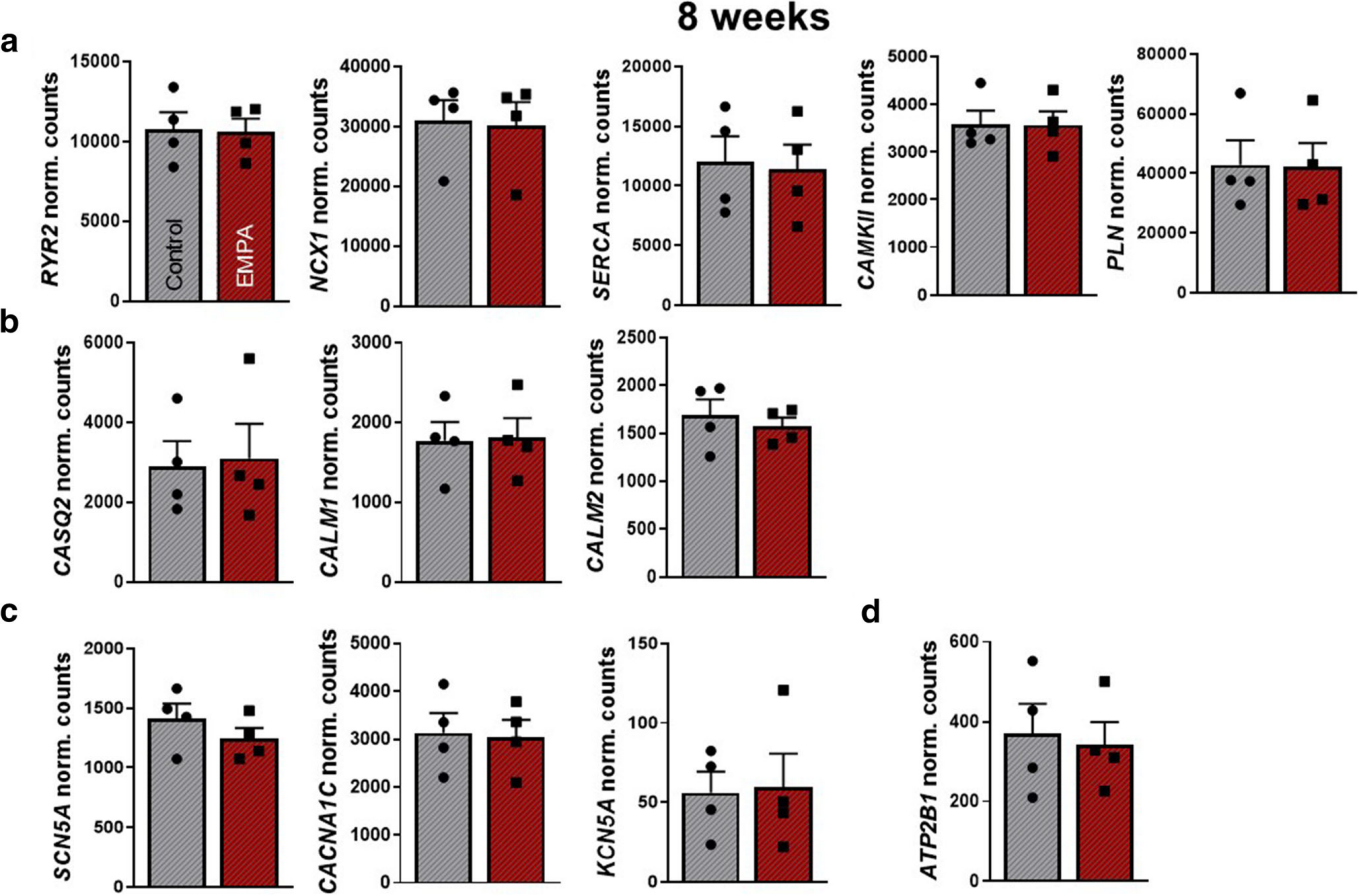
Modulation of gene expression in human-induced pluripotent stem cell cardiomyocytes (iPSC-CM) after chronic (8 weeks) treatment with empagliflozin (0.5 μmol/l, *n* = 4 differentiations). (**a**) Empagliflozin-affected normalized counts of genes as ryanodine-receptor type 2 (*RYR2*), sodium-calcium exchanger (*NCX1*), sarcoplasmic reticulum Ca^2+^ ATPase (*SERCA*), Ca^2+^-/calmodulin-dependent protein kinase II (*CaMKII*), phospholamban (*PLN*), (**b**) calsequestrin 2 (*CASQ2*), calmodulin 1 (*CALM1*), calmodulin 2 (*CALM2*), **(C)** Na^+^ voltage-gated channel alpha subunit 5 (*SCN5A*), Ca^2+^ voltage-gated channel subunit alpha1 C (*CACNA1C*), K^+^ voltage-gated channel subfamily A member 5 (*KCN5A*), (**d**) ATPase plasma membrane Ca^2+^ transporting 1 (*ATP2B1*). For statistical analysis, Student’s *t* test was applied

To further elucidate the molecular mechanisms underlying the pathogenesis of the empagliflozin-dependent phenotype, we performed RNA sequencing of iPSC-CM (4 differentiations from different cell lines from 2 donors), all with and without 0.5 μmol/l empagliflozin exposure for 8 weeks. After normalization for baseline expression, we did not identify differentially expressed genes (DEGs) between the untreated iPSC-CM population and the iPSC-CM after empagliflozin treatment while focusing on important EC-coupling associated genes (Fig. 5a–d). Normalized counts of empagliflozin-treated genes associated with systolic Ca^2+^ rise (*RYR2*), cytoplasmic Ca^2+^ extrusion (*NCX, SERCA, PLN*), cardiac remodeling (*CAMKII*), calcium binding (*CALM1, CALM2, CASQ2),* voltage-gated ion channels (*SCN5A, CACNA1C, KCN5A*), and Ca^2+^ transporting ATPase (*ATP2B1*) are shown in Fig. 5a–d.

### Protein expression of EC-coupling proteins

**Fig. 6:**
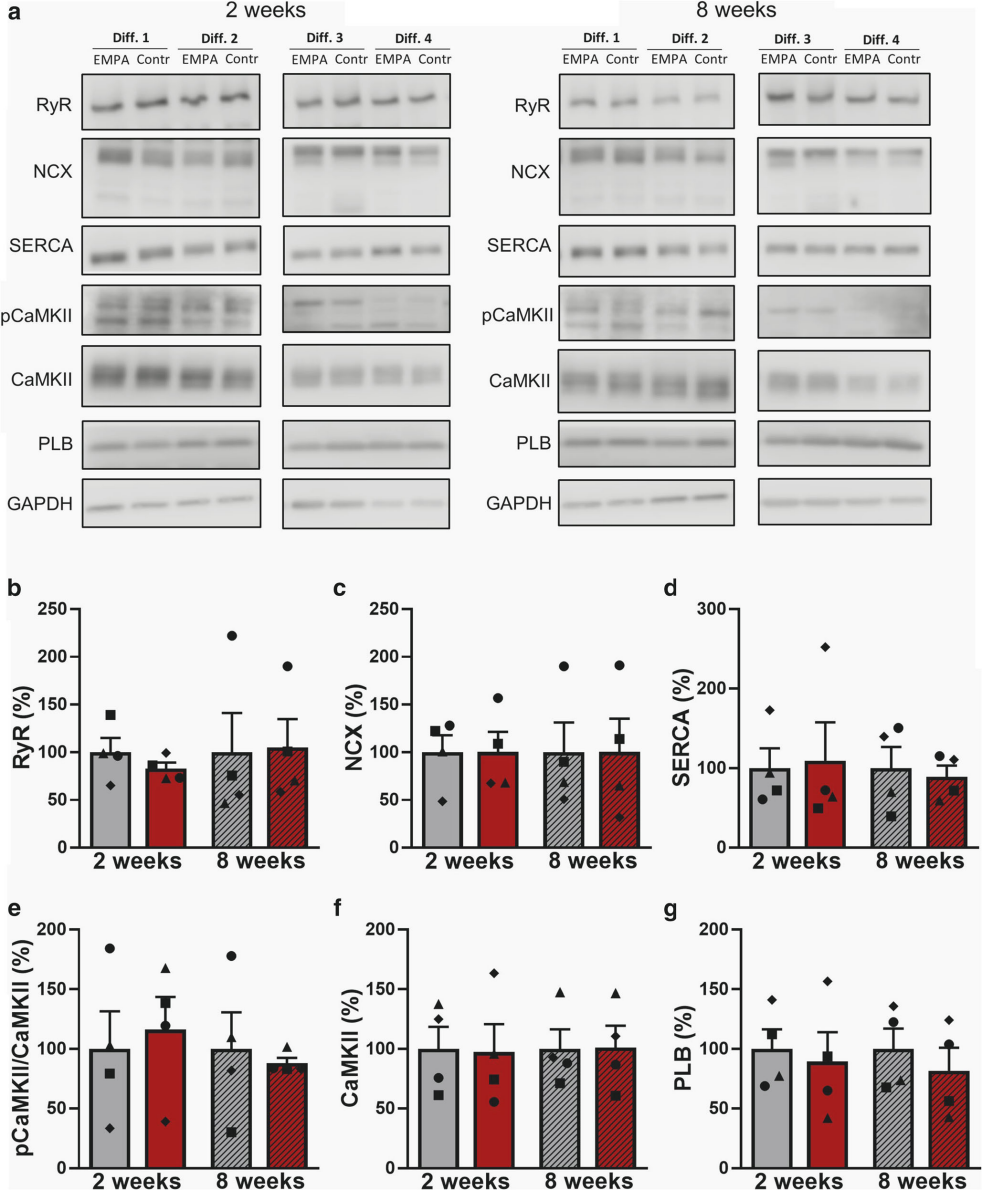
Western blot of EC-coupling proteins in human-induced pluripotent stem cell cardiomyocytes (iPSC-CM). (**a**) Representative original Western blots after treatment with empagliflozin (EMPA) or vehicle control (control) for 2 or 8 weeks from 4 differentiation experiments (Diff.) from 2 healthy donors. GAPDH was used as loading control. (**b**) Mean protein expression levels (normalized to the respective control group at 2 or 8 weeks) in iPSC-CM (*n* = 4 differentiations, matched groups are displayed with matched individual symbols) and effects of 2 and 8 weeks treatment with empagliflozin (EMPA) on ryanodine-receptor type 2 (RyR2), (**c**) sodium-calcium exchanger (NCX), (**d**) sarcoplasmic reticulum Ca^2+^ ATPase (SERCA), (**e**) phosphorylated Ca^2+^-/calmodulin-dependent protein kinase II (pCaMKII), (**f**) Ca^2+^-/calmodulin-dependent protein kinase II (CaMKII), and (**g**) phospholamban (PLB). Groups were statistically analyzed using two-way ANOVA with Sidak’s test for multiple comparisons

To confirm our NGS findings on the protein level, we investigated the chronic effects of empagliflozin on regulatory EC-coupling proteins using Western blots analysis of treated iPSC-CM cultures (4 differentiations from different cell lines from 2 donors). Protein expression levels of EC-coupling proteins did not differ between 2 and 8 weeks. After 2 and 8 weeks of treatment, empagliflozin had no influence on protein expression of RyR2, which determines the systolic rise in [Ca^2+^]_i_ (Fig. 6a, b). Furthermore, SERCA2a, PLB, and NCX, regulating cytosolic Ca^2+^ extrusion, were not affected in iPSC-CM after chronic empagliflozin treatment for 2 or 8 weeks (Fig. 6c, d, g). Also, CaMKII, which directly regulates cardiomyocyte Ca^2+^ cycling and which is strongly associated with adverse cardiac remodeling, was in terms of expression and autophosphorylation not affected in iPSC-CM after chronic in vitro treatment for 2 or 8 weeks (Fig. 6e, f).

## Discussion

This study investigated the direct long-term and thereby clinically relevant effects of empagliflozin on cardiomyocyte EC-coupling and electrophysiology in iPSC-CM. As different mechanisms of SGLT2 inhibitors have been proposed, we aimed to perform blinded treatment, data acquisition, and analysis with a high amount of experimental repetitions. After 2 or 8 weeks of treatment, empagliflozin showed neutral effects on systolic Ca^2+^ transients and SR Ca^2+^ content as well as diastolic Ca^2+^ release. Action potential properties were unchanged upon chronic empagliflozin treatment. Finally, mRNA and protein expression levels of key genes and proteins involved in EC-coupling were not changed by empagliflozin.

Mechanistic evidence is still lacking to fully understand the beneficial clinical effects of SGLT2 inhibitors on cardiovascular and HF outcomes. In particular, the effects of empagliflozin on EC-coupling, which is centrally involved in pathological cardiac remodeling and HF [12], are not fully understood. In order to enhance the translational relevance, this study therefore used human iPSC-CM as a human cardiomyocyte model since the translational potential of animal models is limited. Moreover, utilizing iPSC-CM facilitates large numbers of standardized individual measurements. Of note, we could demonstrate that human iPSC-CM represent a valid translational system for cardiomyocyte function and electrophysiology [13, 20]. To investigate the direct cardiac effects of empagliflozin on cellular EC-coupling and to exclude systemic (secondary) effects on the myocardium (i.e., reduction in pre- and afterload), which could confound our observations, we directly treated iPSC-CM in vitro. Moreover, we performed a chronic treatment for up to 8 weeks in order to better mimic the situation of respective clinical trials, where the beneficial effects of empagliflozin became apparent after ~ 2 months of treatment [1]. Finally, iPSC-CM treatment, data acquisition, and data analysis were performed by externally blinded investigators.

Our data demonstrated that in human iPSC-CM, empagliflozin had neutral effects on systolic Ca^2+^ transients and SR Ca^2+^ content after 2 and 8 weeks of treatment. This result constitutes a safety signal as disturbed Ca^2+^ homeostasis can be associated with arrhythmias and negative inotropy. Moreover, long-term treatment with empagliflozin had neutral effects on action potential properties in human iPSC-CM, which is of relevance for cardiomyocyte electrophysiology as well as clinical safety. According to our findings on systolic Ca^2+^ transients, we previously did also not detect acute effects of empagliflozin on systolic Ca^2+^ transients in isolated CM from patients with HF [7]. However, data from animal cardiomyocyte studies showed an acute reduction of diastolic and systolic [Ca^2+^]_i_ [10], while other experimental animal data showed an increase in Ca^2+^ transient amplitude and SR Ca^2+^ load upon exposure to empagliflozin [9]. Yet, adult CM from either animals or human are barely suitable for long-term treatment protocols, and methodological and species differences may limit the comparison of these studies. Nevertheless, while acute changes (up to 24 h) in Ca^2+^ cycling might be suggested by the aforementioned studies [9, 10], we could here demonstrate that after a treatment duration according to the clinical trial situation, empagliflozin has neutral effects on systolic Ca^2+^ cycling. Of note, it should also be considered that most already established prognostic relevant drugs for treating HF have neutral or even negative effects on systolic Ca^2+^ cycling and inotropy [21]. According to our functional findings, mRNA and protein expression of RyR2 and CaMKII mediating systolic Ca^2+^ release were not affected by chronic empagliflozin treatment. Furthermore, NCX, SERCA, and PLB involved in cytosolic Ca^2+^ extrusion and (for SERCA and PLB) SR Ca^2+^ load were not altered in iPSC-CM after chronic empagliflozin treatment. Thus, these first data on the long-term effects of empagliflozin indicated that, at least in CM, empagliflozin has neutral effects on EC-coupling gene as well as protein expression.

Furthermore, in human iPSC-CM, long-term treatment with empagliflozin did not affect diastolic Ca^2+^ release, which can contribute to cellular arrhythmogenesis and contractile dysfunction [22, 23]. In line with that, RyR2 and CaMKII protein and mRNA expression as well as pCaMKII, which have been shown to strongly regulate diastolic Ca^2+^ sparks [12, 24], were not altered after chronic treatment with empagliflozin in iPSC-CM. However, previous data reported a reduction of diastolic Ca^2+^ sparks as well as CaMKII activity in both human and murine failing CM after 24-h treatment with empagliflozin [9]. In these cells, based on the HF phenotype (while our iPSC-CMs are healthy), a distinct cellular remodeling is present including altered CaMKII activity. Considering the duration of the treatment, acute regulatory stress/oxidative processes may underlie the acute CaMKII-mediated reduction of Ca^2+^ sparks after exposure to empagliflozin in isolated human failing cells [9]. Nevertheless, we think that the treatment duration used in this study is according to the clinical trial situation, the more relevant approach to investigate the effects of empagliflozin.

In our study, we used iPSC-CM from healthy donors. Thus, the transfer of our results for particular cardiovascular diseases like diabetes or heart failure, where cardiac remodeling is present, might however be limited.

As another recently proposed mechanism, inhibitory effects of SGLT2 inhibitors on NHE1 have been discussed [10]. Since NHE1 inhibition has less effects in healthy models [25] and its activity is less in healthy hearts [26], it would be possible that in a healthy model, empagliflozin could have effects on the NHE1 and subsarcolemmal Na^+^/Ca^2+^ homeostasis without greatly changing bulk [Ca^2+^]_i_ in the SR or the cytoplasm. Therefore, in our study, we cannot rule out effects on NHE1 activity. Finally, also other beneficial direct cardiac mechanisms of empagliflozin, i.e., on myofilament function [7] and on inflammatory and oxidative (paracrine) signaling cascades, have been proposed [8, 27, 28], which may serve as a rationale for further mechanistic studies.

In conclusion, we could demonstrate that after a clinically relevant treatment concentration and duration, empagliflozin has neutral effects on cardiomyocyte EC-coupling and electrophysiology in human iPSC-CM. Thus, at least in healthy cardiomyocytes, the beneficial clinical effects of empagliflozin seem to be not mediated by remodeling of cardiomyocyte EC-coupling, which has implications for further mechanistic investigation.

## Data Availability

The datasets generated during and/or analyzed during the current study are available from the corresponding author on reasonable request.
